# NeuroFusion-SLAM: A Deep Neural Network Framework for Real-Time Multi-Sensor SLAM

**DOI:** 10.3390/s26072267

**Published:** 2026-04-07

**Authors:** Chenchen Yu, Wei Wei, Zhihong Cao, Zhiyuan Guo, Bo Fu

**Affiliations:** Shaanxi Key Laboratory for Network Computing and Security Technology, Xi’an University of Technology, Xi’an 710048, China

**Keywords:** multi-sensor fusion, depthwise separable convolution, real-time mapping, Octree mapping, factor graph

## Abstract

While deep learning-based visual SLAM (VSLAM) has achieved remarkable localization accuracy, its high computational cost and latency remain critical bottlenecks for real-time deployment. To address these limitations, this paper presents NeuroFusion-SLAM, a novel multi-sensor fusion framework tailored for both efficiency and robustness. By incorporating depthwise separable convolution, the framework cuts down model parameters by approximately 40% and training time by 49% while preserving localization accuracy, thus boosting real-time inference performance and computational efficiency in large-scale environments. Furthermore, a global edge optimization strategy is proposed by integrating sliding window optimization with a factor graph framework, which effectively improves the global consistency of the system. Extensive experiments on the TUM-VI and KITTI-360 datasets demonstrate that our system achieves real-time performance with an average latency of 30.4 ms per frame. It runs 3× faster than ORB-SLAM2 and 4× faster than VINS-Mono, while maintaining good localization accuracy.

## 1. Introduction

Visual Simultaneous Localization and Mapping (VSLAM) is a core technology in fields such as robotics, autonomous driving, virtual reality (VR), and augmented reality (AR). VSLAM systems utilize one or more visual sensors to build maps and estimate camera positions in real time, making them widely applicable for navigation and localization in complex environments. However, systems relying on a single visual sensor are susceptible to image distortion and feature extraction failures in challenging conditions, such as low light and rapid motion. These limitations can result in localization drift or errors in map construction [1]. Therefore, enhancing the robustness and accuracy of VSLAM systems in such environments remains a critical challenge. Classic feature-based systems such as ORB-SLAM and its extensions have shown strong performance, but their reliance on stable feature extraction and data association can still lead to degraded performance under challenging visual conditions [1,2,3].

In recent years, the rapid advancement of deep learning has introduced new solutions for VSLAM. End-to-end VSLAM systems based on deep neural networks can autonomously learn effective feature representations from raw data, eliminating the dependency on manually designed features common in traditional VSLAM systems. These approaches have shown impressive performance in improving accuracy and robustness, especially in dynamic or texture-deficient environments [4,5,6,7]. Despite their promising results, deep learning-based VSLAM methods often face high computational costs and limited real-time performance [4]. This issue is particularly pronounced in multi-sensor data fusion, where system efficiency becomes a major bottleneck. Representative visual–inertial approaches (e.g., VINS-Mono and DM-VIO) improve robustness by fusing IMU measurements, but achieving both high accuracy and real-time performance in large-scale environments remains challenging [8,9].

In summary, prior studies indicate a practical gap: robustness in dynamic/low-texture scenes, real-time efficiency, and scalability to large environments are often in tension, especially when multi-sensor fusion is involved [4,8,9]. This motivates us to design a VSLAM system that improves robustness while keeping computational overhead bounded, and that supports efficient map storage and querying for large-scale applications [10,11].

To address these challenges, this paper proposes a novel NeuroFusion-SLAM. The system aims to accelerate and optimize the localization and map construction process in large-scale environments by leveraging deep neural networks. Original convolutional layers are replaced with depthwise separable convolution, reducing computational overhead and the number of parameters, thereby enhancing real-time performance and computational efficiency [12,13]. Furthermore, by integrating visual and inertial measurement unit (IMU) sensors, the system employs neural networks to optimize the multi-sensor data fusion process, improving VSLAM performance in dynamic environments. Related multi-sensor fusion SLAM studies have also highlighted the importance of robust fusion under uncertain observations and large-scale settings [10,11].

To further enhance the system’s performance, we propose a global edge optimization strategy based on sliding window optimization and the factor graph framework. Additionally, large-scale 3D map management is streamlined through the use of an Octree data structure, which optimizes storage and querying processes. Since maps produced by SLAM are often consumed by downstream navigation and trajectory planning modules, efficient map processing and querying is also important in practice; for example, dynamic processing of 2D maps has been explored to support robot trajectory planning [14]. Experimental results demonstrate that NeuroFusion-SLAM offers significant advantages over existing methods in multi-sensor fusion, real-time performance, accuracy, and large-scale map management. These improvements make it particularly suitable for complex and large-scale SLAM applications. In summary, the main contributions of this paper are as follows:

- We propose a streamlined neural network architecture integrating depthwise separable convolution and a novel loss function. This design significantly reduces computational redundancy, cutting training time by nearly half while enhancing robustness against dynamic outliers.
- To enable the system to process data in real time with low load, sliding window optimization is integrated into the global factor graph framework; the oldest states are marginalized via the Schur complement to keep the optimization size bounded while maintaining global consistency.
- We introduce an Octree-based data structure for partitioned map management. This approach optimizes memory usage and query efficiency, enabling the system to maintain consistent large-scale 3D dense maps without the memory overflow issues common in dense SLAM.

## 2. Related Works

### 2.1. Visual Simultaneous Localization and Mapping

Since its introduction by Smith and Self in 1986, VSLAM has evolved into a core technology for autonomous driving, robotics, drones, AR and VR. The primary goal of VSLAM is real-time localization and map construction using one or more cameras, without relying on external signals such as GPS. Its key tasks include pose estimation, feature extraction, data association, and map optimization [15].

Representative feature-based systems such as ORB-SLAM/ORB-SLAM2/ORB-SLAM3 have demonstrated accurate and versatile SLAM pipelines with loop closure and map reuse capabilities [1,2,3]. In parallel, direct methods (e.g., LSD-SLAM and Direct Sparse Odometry) estimate motion by minimizing photometric errors and have shown strong performance under sufficient photometric consistency [16,17]. For visual–inertial SLAM/VIO, optimization-based frameworks such as OKVIS, VINS-Mono, DM-VIO, and ORB-SLAM3 further improve robustness by integrating inertial constraints [3,8,9,18].

However, many SLAM systems still face challenges in dynamic or texture-deficient environments, where data association and tracking can degrade, and additional computation is often introduced when extending to multi-sensor fusion and large-scale mapping.

In recent years, deep learning has introduced new solutions for VSLAM. End-to-end VSLAM systems based on deep neural networks autonomously learn feature representations from raw data, eliminating the reliance on manually designed features in traditional VSLAM. These approaches improve accuracy and robustness, especially in dynamic or texture-deficient environments. However, deep learning-based VSLAM systems often face high computational costs and poor real-time performance, particularly in multi-sensor fusion and large-scale environments.

To address these challenges, this paper proposes NeuroFusion-SLAM, a novel multi-sensor fusion VSLAM system designed to accelerate localization and map construction in large-scale environments by leveraging deep neural networks. Depthwise separable convolution replaces original convolutional layers, reducing computational overhead and parameters, thus enhancing real-time performance and efficiency. By integrating visual and inertial measurement unit (IMU) sensors, the system uses neural networks to optimize multi-sensor fusion, improving VSLAM performance in dynamic environments.

A global edge optimization strategy based on sliding window optimization and the factor graph framework is also proposed. Large-scale 3D map management is streamlined with an Octree data structure to optimize storage and querying. Experimental results demonstrate that NeuroFusion-SLAM offers significant advantages in multi-sensor fusion, real-time performance, accuracy, and large-scale map management, making it ideal for complex, large-scale SLAM applications.

### 2.2. Application of Deep Learning in VSLAM

With the rise of deep learning, neural network-based VSLAM has become an active research topic. Deep models can learn image representations and support tasks such as feature extraction, pose estimation and map optimization.

Recent deep SLAM systems such as DROID-SLAM demonstrate strong accuracy and robustness via deep dense optimization modules [4]. Moreover, DBA-Fusion tightly integrates deep dense visual bundle adjustment with multiple sensors in a factor-graph framework, aiming at large-scale localization and mapping [11]. Nevertheless, learning-based components often increase computational demand, which may affect real-time deployment, especially on resource-constrained platforms.

CS-SLAM [7] is a lightweight semantic SLAM method for dynamic scenarios that uses a semantic segmentation network to remove dynamic feature points from images, enhancing SLAM stability and accuracy in dynamic environments. However, its real-time performance in highly dynamic or complex scenarios requires further validation. The Dense Mapping Method [19] integrates YOLOv5 with ORB-SLAM2, improving localization and keyframe selection in dynamic environments. It proposes a keyframe selection method based on inter-frame relative motion to increase accuracy. However, its computational demands may challenge resource-constrained devices. Additionally, in our work, DCT-DSLAM [5], applies Discrete Cosine Transform (DCT) to dynamic SLAM, improving dynamic object detection accuracy through DCT masks. By integrating DCT encoding into the Dyna-SLAM architecture, dynamic masks are converted into low-dimensional vectors, reducing complexity and storage. However, it struggles with rapid changes in dynamic environments, limiting performance under extreme conditions.

Despite deep learning’s progress in visual SLAM, existing deep VSLAM systems face challenges like high computational complexity and poor real-time performance. These limitations hinder their application in resource-constrained environments, especially in large-scale and multi-sensor fusion scenarios, where computational resources and system efficiency are significant obstacles.

### 2.3. Neural Network-Based Multi-Sensor Fusion

To address the limitations of single visual sensors, multi-sensor fusion technology has been extensively studied in VSLAM. By integrating visual sensors with IMUs and GNSS, the robustness, accuracy, and real-time performance of VSLAM systems are significantly enhanced. Multi-sensor fusion provides supplemental localization information when visual data is insufficient, overcoming performance bottlenecks under challenging conditions.

Paper [10] on multi-sensor fusion proposes an IMU-based GNSS simulation neural network that generates location, velocity, and pose data similar to dual-antenna GNSS measurements when GNSS and image data are unavailable. An ESKF fusion network is used to combine GNSS and IMU data, stabilizing the SLAM system. Another paper, Multimask Fusion-Based RGB-D SLAM in Dynamic Environments [6], introduces a dynamic detection method that models feature points through geometric constraints and uses static weight-based pose optimization. By fusing masks from geometric, semantic, and depth data, it identifies dynamic areas for accurate scene reconstruction. While effective with dynamic objects, it struggles to detect completely unknown objects, and the computational burden of mask fusion impacts real-time performance. SLAM technology serves as a core support for autonomous navigation and precise localization of inspection robots. Hu [20] integrated LiDAR and depth camera fusion with SLAM for global navigation and local crack localization. The system, combined with a lightweight semantic segmentation network and hand-eye calibration, achieved submillimeter-level precision in 3D crack detection and spatial positioning.

Although multi-sensor fusion significantly enhances VSLAM systems, several challenges remain. Sensor data often differ in frequency and precision, making alignment and fusion difficult. Additionally, designing a universal framework to process multi-modal sensor data while maintaining real-time performance and robustness is an ongoing research challenge.

The comparison of accuracy, real-time performance, robustness, scalability, and computational demands between traditional SLAM and deep learning-based SLAM is shown in Table 1.

## 3. Materials and Methods

**Figure 1:**
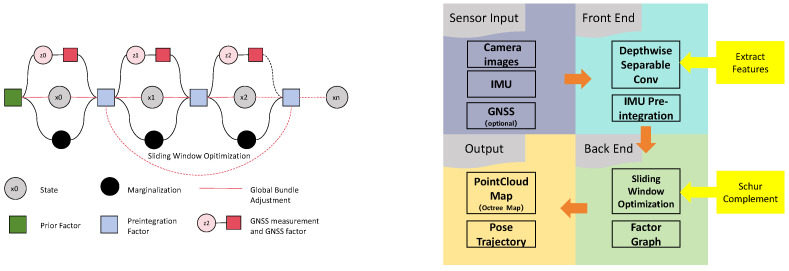
Overall system framework.

NeuroFusion-SLAM is designed as a multi-stage pipeline that addresses three core challenges in visual–inertial SLAM: efficient feature extraction, robust multi-sensor fusion, and scalable map management. The overall system architecture is illustrated in Figure 1, which shows the complete data flow from sensor inputs to optimized pose estimation and map construction. The system operates in three interconnected stages. First, visual features are extracted from camera images using a lightweight neural network based on depthwise separable convolution (Section 3.1), which significantly reduces computational overhead while maintaining feature quality. Second, these visual features are fused with IMU measurements through a global factor graph optimization framework that incorporates sliding window marginalization (Section 3.2), enabling real-time processing in large-scale environments. Finally, the resulting 3D maps are efficiently stored and queried using an Octree-based hierarchical structure (Section 3.3), which prevents memory overflow in long-term operation. Each component is designed to work synergistically: the lightweight feature extractor provides high-quality inputs to the optimization framework, while the efficient map representation supports both real-time localization and downstream applications such as path planning. In the following subsections, we provide detailed descriptions of each component, including the mathematical formulations, algorithmic procedures, and design motivations.

### 3.1. Lightweight Visual Feature Extraction Network

In recent years, with the increasing demand for efficient computation, depthwise separable convolution has become a widely used operation in visual tasks. Depthwise separable convolution significantly reduces the computational load and the number of parameters by decomposing the standard convolution into two separate operations: depthwise convolution and pointwise convolution. Models like MobileNet [13] and Xception [12] have widely adopted depthwise separable convolution in image classification and detection tasks to improve computational efficiency.

In VSLAM, depthwise separable convolution can effectively accelerate image feature extraction and processing, thereby improving the real-time performance and computational efficiency of the SLAM system. This is especially important when dealing with large-scale scenes and multi-sensor fusion tasks, as it significantly reduces computational resource consumption. Regarding the neural network mentioned in DROID-SLAM [4], the authors noted that its training time is relatively long; the train sets are TartanAir [22], requiring a server with four 3090 GPUs to train for a week to achieve the desired results. After our improvements, we are able to achieve the same level of accuracy with just a single 3090 GPU server trained for one week. Additionally, this optimization not only shortens the hardware requirements but also enhances the adaptability of the model to various computing environments, making it more accessible for research institutions and individual developers with limited resources.

**Figure 2:**
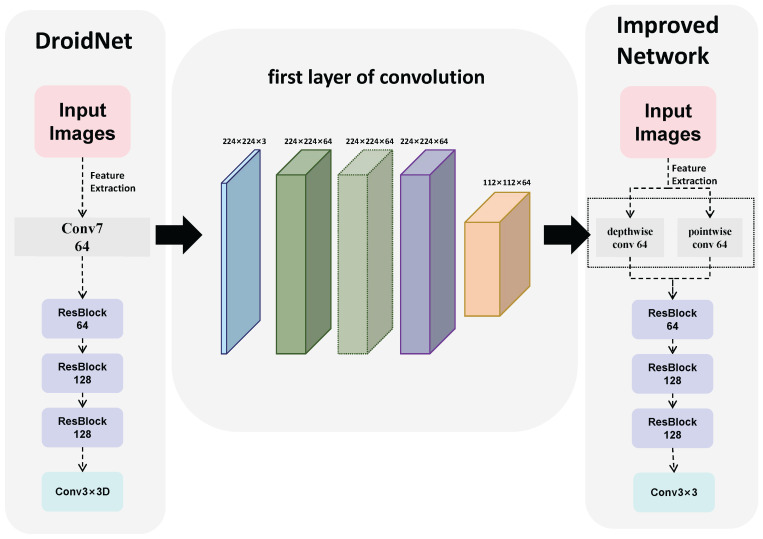
Comparison Diagram of Neural Network Architectures.

The structure of the neural network is shown in the Figure 2.

The image demonstrates how the first convolutional layer is replaced by depthwise convolution (depthwiseconv) and pointwise convolution (pointwiseconv). Additionally, during the training of the neural network, we observed that the loss function for optical flow produced relatively large outputs. We hypothesize that this is due to the presence of many outliers in the optical flow. Inspired by EPE metric, we sum the component-wise losses. Therefore, by combining SmoothL1 Loss, we propose a SmoothPixel loss function to compute the pixel displacement (optical flow) error between two image frames. We found that this approach improves training performance.

(1) $$\begin{array}{cc} \mathrm{SmoothL}1(x) & =\left\{\begin{array}{cc} 0.5x^{2} & \mathrm{if}\;|x|<1\\ |x|-0.5 & \mathrm{if}\;|x|\geq 1 \end{array}\right. \end{array}$$

(2) $$\begin{array}{cc} \mathrm{SmoothPixel} & =\mathrm{SmoothL}1\left(u_{st}-u_{gt}\right)+\mathrm{SmoothL}1\left(v_{st}-v_{gt}\right) \end{array}$$

Let $\left(u_{st},v_{st}\right)$ denote the predicted optical flow (horizontal/vertical components, in pixels) from frame *s* to frame *t* at a pixel and $\left(u_{gt},v_{gt}\right)$ denote the corresponding ground truth flow. SmoothL1 reduces the influence of large residuals by behaving quadratically for small errors and approximately linearly for large errors. This robust regression loss has been commonly used in vision tasks to alleviate the impact of outliers [23,24]. Therefore, SmoothPixel applies SmoothL1 to the horizontal/vertical residuals and sums them to obtain a more robust per-pixel flow error; in training, the final loss is computed by averaging SmoothPixel over all valid pixels.

As shown in Algorithm 1, we illustrate the specific process of lightweight feature extraction and the corresponding robust training strategy. Next, we present the details of the depthwise separable convolution implementation and the SmoothL1 loss-based robust optimization.
**Algorithm 1** Lightweight Network Training with SmoothPixel Loss**Require:** Source image $I_{s}$, Ground Truth Flow $F_{gt}=\left(u_{gt},v_{gt}\right)$ **Ensure:** Optimized Network Parameters θ
  1:**Definitions:**  2:Let F(·;θ) be the feature extraction network.  3:Let DSC(X,K) denote depthwise separable convolution.  4:**while** Training not converged **do**  5:    **// Phase 1: Lightweight Feature Extraction (Innovation 1)**  6:    Initialize feature map $X\leftarrow I_{s}$  7:    **for** each layer *l* in Network **do**  8:        *Step 1.1: Depthwise Convolution (Spatial filtering)*  9:        $X_{dw}\leftarrow \mathrm{DepthwiseConv}\left(X,W_{depth}^{l},\mathrm{groups}=C_{in}\right)$10:        *Step 1.2: Pointwise Convolution (Channel mixing)*11:         $X\leftarrow \mathrm{PointwiseConv}(X_{dw},W_{point}^{l},\mathrm{kernel}=1\times 1)$12:    **end for**13:    *Output Prediction:*14:    $\left(u_{st},v_{st}\right)\leftarrow \mathrm{FlowPredictor}\left(X\right)$15:    **// Phase 2: SmoothPixel Loss Calculation (Innovation 2)**16:    Compute residuals: $\delta_{u}=u_{st}-u_{gt},\;\delta_{v}=v_{st}-v_{gt}$17:    *Define Component-wise SmoothL1 Function:*18:    **for** each component $x\in \{\delta_{u},\delta_{v}\}$ **do**19:        **if** |x|<1 **then**20:            $L_{comp}\left(x\right)\leftarrow 0.5x^{2}$    {Quadratic region for small errors}21:        **else**22:            $L_{comp}\left(x\right)\leftarrow \left|x\right|-0.5$    {Linear region to suppress outliers}23:        **end if**24:    **end for**25:    *Total SmoothPixel Loss:*26:    $L_{total}\leftarrow L_{comp}\left(\delta_{u}\right)+L_{comp}\left(\delta_{v}\right)$27:    **// Phase 3: Back-propagation**28:    Compute gradients $\nabla_{\theta }L_{total}$29:    Update parameters $\theta \leftarrow \theta -\eta \cdot \nabla_{\theta }L_{total}$30:**end while**


Through ablation experiments, we compared two separate improvements made to the original network. The first method replaces standard convolutions with depthwise separable convolution networks, the second method solely adopts our proposed loss function, and the third method combines both innovations. Each configuration was compared against the original neural network model, as detailed in Table 2. Here, EPE measures the Euclidean distance between the predicted flow $\left(u_{st},v_{st}\right)$ and ground truth flow $\left(u_{gt},v_{gt}\right)$, and it is a standard per-pixel flow error. As a standard optical flow evaluation metric, EPE has been widely adopted in benchmark evaluations and the related literature [25].

By analyzing it, we can observe that by introducing smoothPixel and depthwise separable convolution networks, we significantly reduced the training time (by approximately 49%) while simultaneously decreasing the network parameters (by approximately 40%).

The middle part of Figure 2 is a comparison diagram of the first convolutional layer (using the first convolutional layer as an example), where the convolution is divided into depthwise convolution and pointwise convolution. This structure reduces the number of parameters and the computational load of the model while maintaining similar feature extraction capabilities. It allows for a significant reduction in training time while preserving accuracy. On the left side is the DROID-SLAM network structure, and on the right side is our proposed network structure diagram.

The DROID-SLAM system identifies correspondences between frames in an image sequence through feature extraction and correlation calculation. The specific process is as follows. First, for feature extraction, each input image is processed through a network consisting of multiple residual blocks, producing low-resolution feature maps. These feature maps are used to build the correlation volume. Then, a correlation pyramid is constructed. For each pair of frames (i,j) in the frame sequence, the dot product of their feature vectors is computed to generate a 4D correlation volume. (u1,v1) represents a pixel location in the i-th frame. (u2,v2) represents the corresponding pixel location in the *j*-th frame.

This correlation volume is then pooled with average pooling to form a four-level correlation pyramid, as shown in Formula (4).

(3) $$C_{ij}^{u_{1}v_{1}u_{2}v_{2}}=\langle g{\left(I_{i}\right)}^{u_{1}v_{1}},\;g{\left(I_{j}\right)}^{u_{2}v_{2}}\rangle$$

The core of the DROID-SLAM system is a learning-based update operator that combines correlation features, optical flow features, and context features to update the camera’s pose and depth. The specific process is as follows.

1. Feature Injection: The correlation features, optical flow features, and context features are injected into a 3 × 3 convolutional GRU (Gated Recurrent Unit).
2. GRU Update: The convolutional GRU updates its hidden state based on the injected features and generates a corrected optical flow field along with a confidence map.
3. Dense Bundle Adjustment (DBA) Layer: The DBA layer maps the corrected optical flow field to update the camera’s pose and depth, as shown in the following formulas:

(4) $$G_{k+1}=\mathrm{Exp}\left(\Delta \xi \left(k\right)\right)\circ G_{k}$$

(5) $$d_{k+1}=\Delta d\left(k\right)+d_{k}$$

where $G_{k+1}\in SE\left(3\right)$ is the updated camera pose at iteration k+1, $\Delta \xi \left(k\right)\in R^{6}$ is the predicted pose update in the Lie algebra se(3), Exp:se(3)→SE(3) is the exponential map, ∘ denotes the group composition operator on SE(3), $d_{k+1}$ is the updated depth map, and $\Delta d_{k}$ is the predicted depth update.

The exponential map Exp converts a twist (velocity) in the Lie algebra to a rigid transformation in the Lie group, following standard SE(3) parameterization [26].

**Figure 3:**
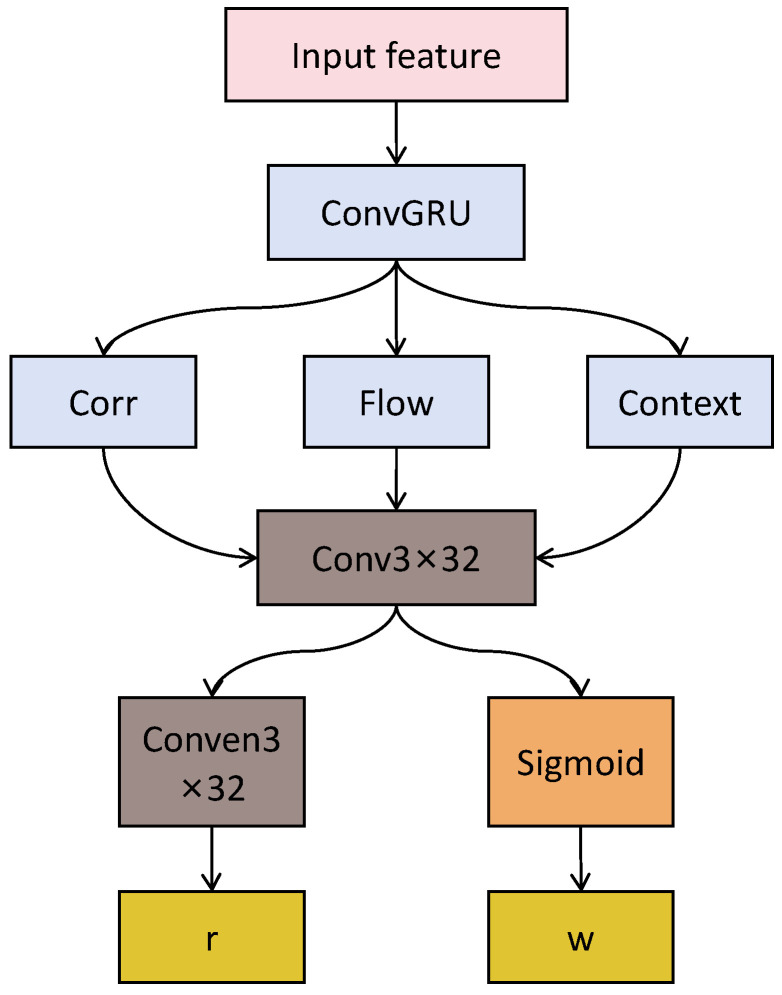
ConvGRU flowchart.

As shown in Figure 3, ConvGRU was used to process time-series data, i.e., Corr (correlation features), Flow (optical flow features), and Context (context features). These features are injected into the ConvGRU to provide additional information that helps the network learn how to update the camera’s pose and depth. Conv3×3 (128) is another convolutional layer used to process the output of the ConvGRU. Conv3×3 (2) is the convolutional layer that outputs two channels and is used to produce the corrected optical flow field. Sigmoid serves as the activation function to transform the convolutional layer’s output into values between 0 and 1, typically used to control flow or provide a probabilistic interpretation.

The corrected optical flow field (r) and confidence map (w) are the final outputs of the ConvGRU. The corrected optical flow field is used to refine the camera pose and depth estimation, while the confidence map provides the reliability of the corrections.

### 3.2. Sliding Window Optimization in Global Factor Graph Framework

Multi-sensor SLAM systems face a fundamental trade-off between global consistency and computational efficiency. Traditional global bundle adjustment optimizes all historical states, ensuring global consistency but suffering from unbounded computational growth in large-scale environments. Conversely, pure sliding window approaches [8,18] maintain fixed computational complexity but discard old states entirely, leading to accumulated drift and inconsistent maps. We propose a hybrid approach that combines the strengths of both paradigms: sliding window optimization maintains real-time performance by limiting the active optimization window, while marginalization via the Schur complement preserves information from discarded states as prior constraints in the global factor graph.

The sliding window contains a fixed number of recent frames, which are represented as nodes in the factor graph. As new frames are added, the oldest frames are removed, and the window slides forward. Let the window size be N, then at any given time, the optimization problem only involves the N frames within the window. Afterward, marginalization is required. When the i-th frame is removed, the pose and depth states of that frame need to be marginalized from the factor graph. The marginalization operation is implemented using the Schur complement; it transforms the constraints from removed frames into a prior factor. This preserves the accumulated information of the system’s history, effectively acting as a global constraint that prevents the current sliding window from decoupling from the global trajectory, thereby maintaining long-term consistency. The specific formulas are as follows:

(6) $$H_{\mathrm{marg}}=H-H_{i}C_{i}^{-1}H_{i}^{T}$$

(7) $$v_{\mathrm{marg}}=v-H_{i}C_{i}^{-1}v_{i}$$

(8) $$\min_{x}\sum_{i}\rho \left(r_{i}\left(x\right)\right)$$

where *H* and *v* are the global Hessian matrix and information vector, respectively, and $H_{i}$ and $v_{i}$ are the portions related to frame *i*. In the factor graph optimization framework, our goal is to minimize the total sum of all measurement residuals. Each factor represents a specific measurement or prior knowledge, and they collectively define a nonlinear least squares problem. The optimization problem can be expressed as Formula (8), where *x* is the set of state variables, including the camera pose, 3D point positions, IMU biases, etc.; $r_{i}\left(x\right)$ is the residual of the *i*-th factor; and ρ is the penalty function. In this case, the Huber loss function is chosen to improve robustness.

The IMU pre-integration factor takes into account the IMU measurements of acceleration and angular velocity and transforms them into position and orientation variables. Let Δt be the time interval between two consecutive IMU measurements and α and ω represent the measured acceleration and angular velocity, respectively. The integrated change in pose can be expressed as follows:

(9) $$\begin{array}{cc} \Delta R & =\mathrm{Exp}\left(\frac{1}{2}\omega \Delta t\right)\\ \Delta p & =v\Delta t+\frac{1}{2}a\Delta t^{2} \end{array}$$

where *R* and *p* are the initial orientation and position, respectively, and v is the initial velocity. The IMU pre-integration factor is represented as shown in Formula (12):

(10) $$r_{IMU}\left(x_{k},x_{k+1}\right)=\left[\begin{array}{c} R_{k+1}^{T}\left(p_{k+1}-p_{k}-\nu_{k}\Delta t+\frac{1}{2}a\Delta t^{2}\right)\\ \omega_{k}\Delta t-\left(R_{k+1}R_{k}^{T}-I\right) \end{array}\right]$$

where $x_{k}={\left[p_{k},v_{k},R_{k},b_{a},b_{g}\right]}^{T}$ represents the state vector, including position $p_{k}$, velocity $v_{k}$, orientation $R_{k}$, and the biases of the accelerometer and gyroscope $b_{a},b_{g}$.

The prior information factor provides the system with an initial estimate and uncertainty. If we know the initial pose of the system, we can define it as

(11) $$r_{\mathrm{prior}}\left(x_{0}\right)=x_{0}-x_{\mathrm{prior}}$$

(12) $$H_{\mathrm{prior}}=I$$

where $x_{prior}$ is the prior state, and I is the identity matrix. The bundle adjustment factor optimizes the camera pose and 3D point positions by minimizing the reprojection error. For each observed feature point j and the corresponding camera pose i, the BA factor can be expressed as

(13) $$r_{\mathrm{BA}}\left(X,P\right)=P_{\mathrm{cam}}X-z$$

(14) $$H_{\mathrm{BA}}=\left[\frac{\partial P_{\mathrm{cam}}}{\partial x}\;\frac{\partial P_{\mathrm{cam}}}{\partial P}\right]$$

where X is the 3D point position, P is the camera pose, z is the observed point in the image, and $P_{\mathrm{cam}}$ is the camera projection matrix.

The GNSS factor provides position information in the global reference frame. For the position $P_{gnss}$, the GNSS factor can be expressed as

(15) $$\begin{array}{cc} r_{\mathrm{gnss}} & =T_{\mathrm{gnss}}x-P_{\mathrm{gnss}}\\ H_{\mathrm{gnss}} & =\frac{\partial T_{\mathrm{gnss}}}{\partial x} \end{array}$$

where $P_{\mathrm{cam}}$ is the transformation matrix from the GNSS coordinate system to the local coordinate system.

Solving this system gives the update for the state variables Δx, and then the state variables are updated as follows:

(16) $$x^{*}=x+\Delta x$$

By integrating data from multiple sensors in the above manner, precise state estimation and localization are achieved.

### 3.3. Efficient Map Management via Octree Structure

In VSLAM, map management is a critical issue [2]. On one hand, as the exploration area expands, the map continuously grows, and it is necessary to control the map size to ensure real-time performance. On the other hand, the map contains a large amount of information, and it is important to organize and utilize this information efficiently to improve both mapping and localization accuracy [19]. Dense SLAM systems like ElasticFusion [27] and DTAM [28] produce rich 3D reconstructions but suffer from memory overflow in long-term operation: memory consumption grows linearly with the explored area, eventually exceeding available resources. While sparse SLAM systems (e.g., ORB-SLAM) avoid this issue by storing only landmark points, they lack the dense geometric information required by downstream tasks such as obstacle avoidance and path planning. To address this, we adopt an Octree-based hierarchical map representation [29] that adaptively partitions the 3D space. Unlike uniform grids, Octrees allocate memory only where needed: high-resolution voxels are for detailed regions and coarse voxels for empty space. This adaptive resolution enables efficient storage (reducing memory by 60–80% compared to uniform grids in our experiments) and fast spatial queries (logarithmic complexity for point insertion and nearest-neighbor search). Moreover, the hierarchical structure naturally supports multi-resolution map management, which is essential for real-time planning in large-scale environments. During the octree node insertion process, for a given node P with coordinates (x,y,z), we determine its child node index based on the following rules:

(17) $$\mathrm{Index}=\left(\frac{x>centerx}{2}\right)*4+\left(\frac{y>centery}{2}\right)*2+\left(\frac{z>centerz}{2}\right)$$

Here, ’center’ represents the center point of the spatial volume. If the coordinates of node P are greater than the corresponding coordinates of the center point, the corresponding bit is set to 1; otherwise, it is set to 0. In this way, we obtain an index value ranging from 0 to 7, which is used to identify the child nodes.

## 4. Experiments and Results

We tested our system on the public TUM-VI [30] and KITTI-360 [31] datasets, along with several other algorithms, demonstrating excellent mapping and tracking performance.

We used a sparse matrix library to reduce memory usage and computational complexity for real-time processing. Incremental updates were employed to avoid recomputing the entire system, and GPU acceleration was utilized for large-scale matrix operations to improve computational efficiency. The experimental platform was a workstation running Ubuntu 20.04 LTS. The deep learning models were implemented using PyTorch 1.12 with CUDA 11.3. The hardware configuration, as detailed in Table 3, features an Intel Xeon 8336C CPU and an NVIDIA RTX 3090 GPU.

### 4.1. Comprehensive Evaluation on the TUM-VI Dataset

First, we conducted tests on the public TUM-VI dataset. The TUM-VI (Technical University of Munich - Visual Inertial) dataset is specifically designed for research on visual–inertial odometry (VIO). It is provided by the Technical University of Munich (TUM) and aims to promote the development of autonomous navigation technologies for mobile platforms, such as robots and drones, in various environments. The TUM-VI dataset contains rich sensor data, including stereo camera images, IMU (inertial measurement unit) data, and high-precision ground truth trajectories. It consists of a total of 28 sequences, covering various challenges that may be encountered in the practical application of visual–inertial navigation. These data were collected in various indoor and outdoor scenes, reflecting different lighting conditions, texture variations, and the effects of dynamic objects. A notable feature of this dataset is its diversity and complexity. It not only covers linear and rotational motion but also includes challenging maneuvers such as fast movements and abrupt stops.

For the evaluation, we selected the Absolute Trajectory Error (ATE) as the metric, which refers to the difference between the estimated trajectory and the ground truth trajectory (i.e., the actual trajectory). The specific results for our algorithm, along with ORB-Mono, VINS-Mono [8], DROID-Mono, DM-VIO [9] and DBA-Fusion [11], are shown in Table 4. NeuroFusion-SLAM outperforms competing methods in most sequences. This superior performance can be attributed to two key factors.

1. Robustness in texture-deficient scenes: In corridor environments like Corr1 and Corr3, traditional feature-based methods often suffer from tracking loss due to insufficient distinctive features. In contrast, our deep neural network, enhanced by depthwise separable convolution, extracts high-level semantic features that remain robust even in low-texture regions.
2. Effective fusion strategy: Compared to VINS-Mono, our system leverages the confidence map (generated in Section 3.1) to dynamically weight visual residuals during the factor graph optimization. This allows the system to trust IMU predictions more when visual reliability drops, leading to better trajectory smoothness.

From the previous Table 4, we can analyze and roughly categorize the dataset into three types. One category is Corr1-3, which represents long corridor environments, where the impact of dynamic objects is significantly reduced compared to outdoor environments (Outd3-4). We can see that the ATE for all algorithms is noticeably lower in the long corridor environment. On the other hand, in outdoor environments with dynamic objects and rapid movements, ORB-Mono, VINS-Mono, and DROID-Mono show much higher ATE values compared to the other two algorithms. Overall, our improved algorithm achieves the lowest ATE in most of the sequences within this dataset.

### 4.2. Comprehensive Evaluation on the KITTI-360 Dataset

The next test was conducted on the KITTI-360 dataset. The KITTI-360 dataset is a comprehensive, large-scale 3D environment perception dataset jointly released by Karlsruhe Institute of Technology (KIT) and Toyota Technological Institute. It aims to provide a rich resource for autonomous driving and computer vision research. The dataset includes high-resolution stereo camera images, 360-degree panoramic LiDAR scan data, high-precision GPS/IMU data, as well as pixel-level semantic and instance segmentation annotations. It covers a wide range of urban driving scenarios and weather conditions, offering more than 43,000 frames of image and LiDAR scan data.

For the test metrics, we selected the relative translation error and the relative rotation error, as shown in Table 5. The relative translation error refers to the relative error between the estimated trajectory’s translation component and the ground truth trajectory’s translation component over a certain time period. The calculation formula is as follows:

(18) $$t_{rel}=\frac{∥t_{est}-t_{gt}∥}{d}\times 100\%$$

The numerator represents the difference between the estimated position and the ground truth position, while d represents the distance traveled during that period. The final result is expressed as a percentage (%).

The relative rotation error $r_{\mathrm{rel}}=\frac{\theta }{d}$ refers to the relative difference between the rotational components of the estimated trajectory and the groundtruth trajectory over a certain time period, where θ denotes the rotation angle error between the estimated and ground truth relative rotations over the same segment (in degrees), and *d* is the traveled distance of that segment (in meters). Following the standard KITTI odometry evaluation protocol, both metrics are calculated by averaging errors over subsequences of varying lengths (100 m to 800 m).

Table 5 presents a quantitative comparison of our method against state-of-the-art baselines, categorizing them into geometry-based methods, such as ORB-SLAM2 and VINS-Mono, and learning-based approaches, such as Droid-SLAM and DBA-Fusion. As illustrated in the results, our proposed method achieves competitive or superior performance across most sequences. Specifically, compared to traditional geometry-based systems, our approach demonstrates a significant reduction in translation error, particularly in Sequences 0000 and 0002, indicating effective mitigation of cumulative drift over long trajectories. Regarding learning-based competitors, while DBA-Mono shows strong performance in specific sequences, our method maintains better overall stability with consistently lower rotation errors. It is worth noting that Droid-Mono exhibits anomalously high translation errors, which is likely attributed to the severe scale drift and ambiguity inherent in pure monocular learning methods on this dataset. In contrast, the lower errors achieved by our method highlight its superior scale consistency and robustness in complex urban environments.

### 4.3. Trajectory Point Cloud Map and Real-Time Performance

**Figure 4:**
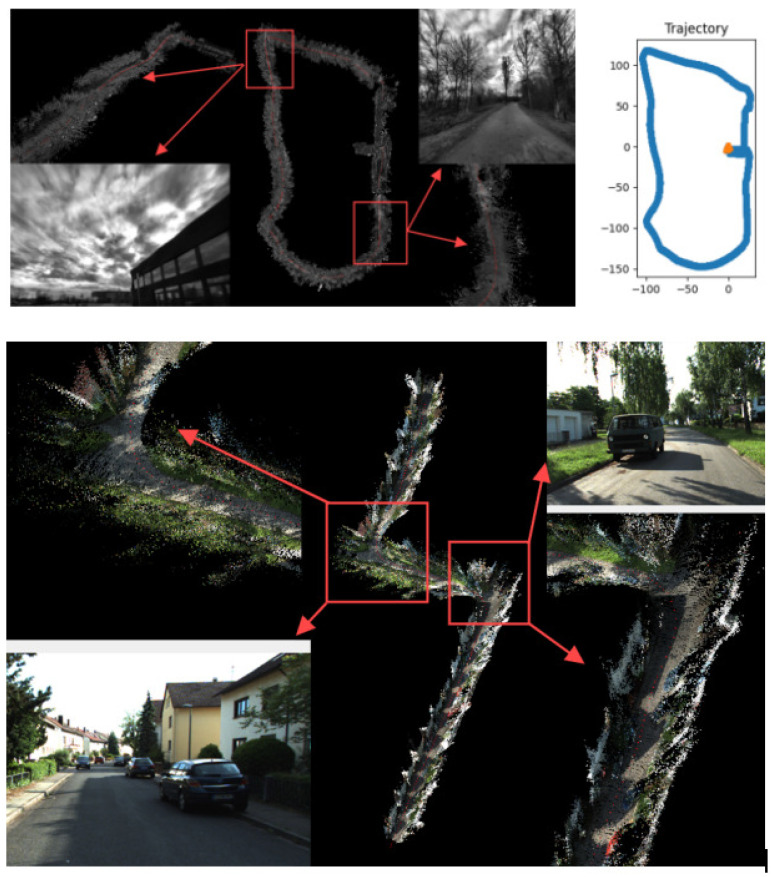
Mapping performance and trajectory tracking.

The NeuroFusion-SLAM system ultimately generates a three-dimensional point cloud map. Figure 4 is divided into two main sections: upper and lower. The upper section uses the ‘outdoors’ sequence from the TUM-VI dataset, while the left side shows the real-time operation and the generation of map points, and the right side shows the trajectory tracking. The lower section features the ‘0000’ sequence from the KITTI-360 dataset, illustrating the real-time mapping of the corresponding color images.

**Figure 5:**
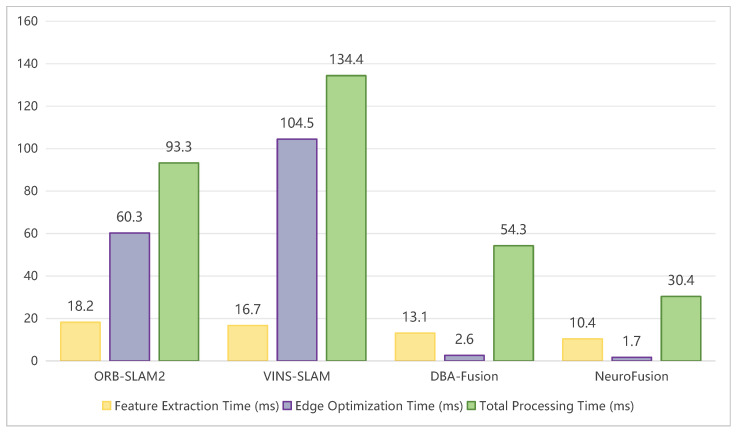
Visualization of computational efficiency and runtime on the TUM-VI dataset.

Regarding the real-time performance of various methods as shown in Table 6 and Figure 5, we conducted comparisons within the TUM-VI dataset. We compared the algorithms in terms of feature extraction, edge optimization, and program runtime. In each category of data, our algorithm consistently showed the least amount of time. During the design of NeuroFusion-SLAM, special consideration was given to lightweight architecture to accommodate environments with limited resources. By optimizing the network structure and reducing the number of parameters, our framework is able to reduce the demand for computational resources while maintaining accuracy. Specifically, the proposed method attained an average total processing time of 30.4 ms, which is approximately three times faster than ORB-SLAM2 (93.3 ms) and more than four times faster than VINS-SLAM (134.4 ms). This substantial improvement ensures real-time performance even on embedded platforms, enabling its application in mobile robotics and augmented reality.

Edge optimization time specifically measures the average time consumption of the single-frame factor graph update process, which includes the neural network-based residual computation and the incremental marginalization via the Schur complement. This process runs on the GPU (RTX 3090, as detailed in Table 3). This metric is distinct from the macroscopic system frame rate. Instead, it serves to evaluate the efficiency of our neural constraint generation and solving module against traditional CPU-based sparse bundle adjustment solvers used in ORB-SLAM2 and VINS-Mono. ation Time as reported in Table 6 specifically measures the average time consumption of the single-frame factor graph update process, which includes the neural network-based residual computation and the incremental marginalization via the Schur complement. This process runs on the GPU (RTX 3090, as detailed in Table 3). This metric is distinct from the macroscopic system frame rate. Instead, it serves to evaluate the efficiency of our neural constraint generation and solution module against traditional CPU-based sparse bundle adjustment solvers used in ORB-SLAM2 and VINS-Mono.

## 5. Conclusions

This paper presented NeuroFusion-SLAM, a computationally efficient framework for multi-sensor simultaneous localization and mapping. By replacing standard convolutions with depthwise separable convolutions, we reduced the network training time by 49% and parameter count by 40%. The integration of a SmoothPixel loss and sliding window factor graph optimization enabled the system to robustly handle dynamic outliers. Quantitative evaluations on TUM-VI and KITTI-360 datasets showed that NeuroFusion-SLAM outperforms state-of-the-art geometry-based methods in efficiency, achieving a total processing time of 30.4 ms vs. 93.3 ms for ORB-SLAM2. Furthermore, NeuroFusion-SLAM demonstrates competitive localization accuracy while enabling dense mapping in large-scale environments.

In future work, we will further enhance robustness in outdoor environments with stronger dynamics and illumination changes by improving dynamic-object handling and correspondence reliability. We also plan to extend the current multi-sensor factor-graph framework with more adaptive factor weighting and marginalization strategies to better cope with varying sensor quality and motion patterns. Finally, we will optimize the model and the overall pipeline for embedded deployment (e.g., model compression/quantization) and validate scalability on larger and more diverse datasets.

## Figures and Tables

**Table 1:** Comparison of geometry-based and learning-based SLAM.

Characteristic	Geometry-Based	Learning-Based
Accuracy (typical scenes)	static/well-textured [1,3,17]	dynamic/complex scenes [4,6,7]
Real-time Performance	efficient/lightweight [1,8]	often model-limited [4,11]
Robustness	may degrade in challenging conditions [1,16]	often stronger in complex environments [6,7,21]
Scalability	mature pipelines/multimap [3]	design-dependent (computation and fusion) [10,11]
Computational Demand	low–medium [1,8]	often high [4,11]

Note: This table summarizes qualitative tendencies rather than absolute conclusions.

**Table 2:** Ablation study of DROID-NET with different configurations.

Model Configuration	Loss Function	Time (h)	Params	Performance
Original DROID-NET	Original Loss Function	672	10 M	AEPE: 1.51, Acc: 85%
Only DSC ^1^	Original Loss Function	315	6 M	AEPE: 1.40, Acc: 86%
Only Modified Loss	New Optical Flow Loss	701	10 M	AEPE: 1.30, Acc: 88%
Both Modifications	New Optical Flow Loss	342	6 M	AEPE: 1.15, Acc: 92%

^1^ DSC refers to depthwise separable convolution. Original DROID-NET refers to the official implementation and pre-trained weights provided by Teed [4].

**Table 3:** Computer configuration.

Type	Mode
CPU	Xeon 8336C
System Memory	128 GB
Video Memory	24 GB
Solid State Devices	1 TB
Video Card	NVIDIA RTX 3090

**Table 4:** Complete ATE results on all TUM-VI sequences.

Sequence	ORB-Mono	VINS-Mono	DROID-Mono	DBA-Fusion	Ours
Indoor Corridors					
Corridor1	5.24	3.43	4.29	0.57	**0.21**
Corridor2	6.89	4.59	4.35	0.78	**0.46**
Corridor3	3.69	3.45	4.04	0.54	**0.51**
Indoor Rooms					
Room3	9.21	6.32	6.94	1.45	**1.02**
Room4	8.67	5.89	6.43	1.31	**0.93**
Magistrale3	13.42	9.67	10.54	2.34	**1.89**
Magistrale4	12.15	8.76	9.78	2.12	**1.71**
Outdoor Environments					
Outdoors3	41.24	32.43	40.38	**8.17**	10.40
Outdoors4	23.43	15.29	22.47	4.84	**3.77**
Average	13.77	9.98	12.14	2.46	2.32

The bold text refer to the best-performing results among all comparable experiments.

**Table 5:** Relative translation error and relative rotation error for various algorithms on different sequences in the KITTI-360 dataset.

Seq.	ORB Mono		VINS Mono		Droid Mono		DBA Mono		Ours Mono	
	$t_{\mathrm{rel}}$	$r_{\mathrm{rel}}$	$t_{\mathrm{rel}}$	$r_{\mathrm{rel}}$	$t_{\mathrm{rel}}$	$r_{\mathrm{rel}}$	$t_{\mathrm{rel}}$	$r_{\mathrm{rel}}$	$t_{\mathrm{rel}}$	$r_{\mathrm{rel}}$
0000	2.378	0.123	1.895	0.180	18.023	0.224	0.680	0.114	**0.625**	**0.101**
0002	1.311	0.217	1.010	0.195	8.052	0.277	0.651	0.175	**0.634**	**0.145**
0003	7.054	0.158	2.771	0.091	20.221	0.206	**1.019**	0.103	1.123	**0.099**
0004	1.955	0.207	1.706	0.187	13.559	0.479	**0.556**	**0.152**	0.712	0.169
0005	1.422	0.235	1.179	0.221	9.223	0.401	0.620	0.208	**0.459**	**0.158**

The bold text refer to the best-performing results among all comparable experiments.

**Table 6:** Comparison of real-time performance of various methods on the TUM-VI dataset.

Method	Feature Extraction Time (ms)	Edge Optimization Time (ms)	Total Processing Time (ms)
ORB-SLAM2	18.2	60.3	93.3
VINS-SLAM	16.7	104.5	134.4
DBA-Fusion	13.1	2.6	54.3
NeuroFusion	10.4	1.7	30.4

## Data Availability

The data that support the findings of this study are available in TUM-VI [30] and KITTI-360 [31].

## References

[1] Mur-Artal R., Tardós J.D. (2017). ORB-SLAM2: An Open-Source SLAM System for Monocular, Stereo, and RGB-D Cameras. IEEE Trans. Robot..

[2] Mur-Artal R., Montiel J.M.M., Tardós J.D. (2015). ORB-SLAM: A Versatile and Accurate Monocular SLAM System. IEEE Trans. Robot..

[3] Campos C., Elvira R., Rodríguez J.J.G., Montiel J.M.M., Tardós J.D. (2021). ORB-SLAM3: An Accurate Open-Source Library for Visual, Visual–Inertial, and Multimap SLAM. IEEE Trans. Robot..

[4] Teed Z., Deng J. (2021). DROID-SLAM: Deep Visual SLAM for Monocular, Stereo, and RGB-D Cameras. Proceedings of the 35th International Conference on Neural Information Processing Systems.

[5] Fu B., Guo Z. (2024). DCT-DSLAM: Discrete Cosine Transform Towards the DYNAMIC SLAM. 2024 9th International Symposium on Computer and Information Processing Technology (ISCIPT).

[6] Gao Y., Hu M., Chen B., Yang W., Wang J., Wang J. (2024). Multimask Fusion-Based RGB-D SLAM in Dynamic Environments. IEEE Sens. J..

[7] Guo Z., Dong N., Zhang Z., Mai X., Li D. (2024). CS-SLAM: A lightweight semantic SLAM method for dynamic scenarios. IEEE Trans. Cogn. Dev. Syst..

[8] Qin T., Li P., Shen S. (2018). VINS-Mono: A Robust and Versatile Monocular Visual-Inertial State Estimator. IEEE Trans. Robot..

[9] Stumberg L.V., Cremers D. (2022). DM-VIO: Delayed Marginalization Visual-Inertial Odometry. IEEE Robot. Autom. Lett..

[10] Feng M., Yi X., Wang K., Cheng Z. (2024). Multi-sensor fusion visual SLAM for uncertain observations. 2024 5th International Conference on Computer Vision, Image and Deep Learning (CVIDL).

[11] Zhou Y., Li X., Li S., Wang X., Feng S., Tan Y. (2024). DBA-Fusion: Tightly Integrating Deep Dense Visual Bundle Adjustment With Multiple Sensors for Large-Scale Localization and Mapping. IEEE Robot. Autom. Lett..

[12] Chollet F. (2017). Xception: Deep Learning with Depthwise Separable Convolutions. arXiv.

[13] Howard A.G., Zhu M., Chen B., Kalenichenko D., Wang W., Weyand T., Andreetto M., Adam H. (2017). MobileNets: Efficient Convolutional Neural Networks for Mobile Vision Applications. arXiv.

[14] Azubairi S., Petunin A., Alwan H.L., Msallam M.M., Humaidi A.J. (2025). Dynamic Processing 2D Maps Method for Robot’s Trajectory Planning. Proc. Eng. Technol. Innov..

[15] Fu B., Guo Z., Wei W., Ren Y. (2024). Optimization of Visual SLAM for Mobile Robots in Complex Environments: Tight Coupling of Visual-Inertial Fusion and Efficient Loop Closure Detection Strategies. 2024 3rd International Conference on Robotics, Artificial Intelligence and Intelligent Control (RAIIC).

[16] Engel J., Schöps T., Cremers D. (2014). LSD-SLAM: Large-Scale Direct Monocular SLAM. European Conference on Computer Vision (ECCV).

[17] Engel J., Koltun V., Cremers D. (2018). Direct Sparse Odometry. IEEE Trans. Pattern Anal. Mach. Intell..

[18] Leutenegger S., Lynen S., Bosse M., Siegwart R., Furgale P. (2015). Keyframe-based visual–inertial odometry using nonlinear optimization. Int. J. Robot. Res..

[19] Kan X., Shi G., Yang X. (2024). Dense Mapping Method for Indoor Dynamic Scenes Using an Improved ORB-SLAM2 Algorithm Based on RGB-D. 2024 IEEE 25th China Conference on System Simulation Technology and its Application (CCSSTA).

[20] Hu K., Chen Z., Kang H., Tang Y. (2024). 3D vision technologies for a self-developed structural external crack damage recognition robot. Autom. Constr..

[21] Bescós B., Fácil J.M., Civera J., Neira J. (2018). DynaSLAM: Tracking, Mapping, and Inpainting in Dynamic Scenes. IEEE Robot. Autom. Lett..

[22] Wang W., Zhu D., Wang X., Hu Y., Qiu Y., Wang C., Hu Y., Kapoor A., Scherer S. (2020). TartanAir: A Dataset to Push the Limits of Visual SLAM. 2020 IEEE/RSJ International Conference on Intelligent Robots and Systems (IROS).

[23] Girshick R. (2015). Fast R-CNN. 2015 IEEE International Conference on Computer Vision (ICCV).

[24] Huber P.J. (1964). Robust Estimation of a Location Parameter. Ann. Math. Stat..

[25] Baker S., Scharstein D., Lewis J.P., Roth S., Black M.J., Szeliski R. (2011). A Database and Evaluation Methodology for Optical Flow. Int. J. Comput. Vis..

[26] Solà J., Deray J., Atchuthan D. (2018). A micro Lie theory for state estimation in robotics. arXiv.

[27] Whelan T., Salas-Moreno R.F., Glocker B., Davison A.J., Leutenegger S. (2016). ElasticFusion: Real-time dense SLAM and light source estimation. Int. J. Robot. Res..

[28] Newcombe R.A., Lovegrove S.J., Davison A.J. (2011). DTAM: Dense tracking and mapping in real-time. 2011 International Conference on Computer Vision.

[29] Hornung A., Wurm K.M., Bennewitz M., Stachniss C., Burgard W. (2013). OctoMap: An efficient probabilistic 3D mapping framework based on octrees. Auton. Robot..

[30] Schubert D., Goll T., Demmel N., Usenko V., Stückler J., Cremers D. (2018). The TUM VI Benchmark for Evaluating Visual-Inertial Odometry. 2018 IEEE/RSJ International Conference on Intelligent Robots and Systems (IROS).

[31] Liao Y., Xie J., Geiger A. (2023). KITTI-360: A Novel Dataset and Benchmarks for Urban Scene Understanding in 2D and 3D. IEEE Trans. Pattern Anal. Mach. Intell..

